# Immunohistochemical Expression of Tyrosine Kinase Receptors EGFR and CD117 in Canine Urothelial Carcinoma

**DOI:** 10.3390/vetsci13100999

**Published:** 2026-09-23

**Authors:** Rita Files, Catarina Nunes, Maria Cardoso, Maria Silva, Leonor Delgado, Justina Prada, Isabel Pires

**Affiliations:** 1 Department of Veterinary Sciences, University of Trás-os-Montes and Alto Douro, 5000-801 Vila Real, Portugaljprada@utad.pt (J.P.); 2 Department of Biology and Environment, School of Life and Environmental Sciences, University of Trás-os-Montes and Alto Douro, 5000-801 Vila Real, Portugal; 3 UNIPRO—Oral Pathology and Rehabilitation Research Unit, University Institute of Health Sciences—CESPU (IUCS-CESPU), 4585-116 Gandra, Portugal; 4 Department of Pathology, INNO Specialized Veterinary Services, 4710-503 Braga, Portugal; 5 Animal and Veterinary Research Centre (CECAV), Associate Laboratory for Animal and Veterinary Sciences (AL4AnimalS), University of Trás-os-Montes and Alto Douro, 5000-801 Vila Real, Portugal

**Keywords:** bladder cancer, dogs, immunohistochemistry, tumor aggressiveness, c-KIT, EGFR, translational medicine

## Abstract

Canine urothelial carcinoma is the most common malignant neoplasm of the lower urinary tract in dogs and is characterized by aggressive biological behavior, high invasiveness, and metastatic potential. Despite advances in our understanding of this neoplasm, few molecular biomarkers with diagnostic, prognostic, and therapeutic value have been identified. In this context, the present study aimed to evaluate the immunohistochemical expression of the tyrosine kinase receptors EGFR (Epidermal Growth Factor Receptor) and CD117 (c-KIT) in canine urothelial carcinomas. EGFR and CD117 were positive in the majority of cases; both were individually associated with a high histologic grade. In multivariate analysis, a high histological grade was independently associated with EGFR, whereas CD117 was not. In a separate model, EGFR/CD117 co-expression was also independently associated with high histological grade. EGFR and CD117 expression were themselves significantly associated, with 95.0% of EGFR-positive tumors also CD117-positive, a pattern that warrants further investigation into a potential interplay between these pathways during urothelial carcinogenesis. To our knowledge, this is the first study to simultaneously assess EGFR and CD117 in canine urothelial carcinoma; these findings support further investigation into their potential as combined therapeutic targets and suggest that the canine model may offer valuable insights for human precision oncology.

## Simple Summary

Canine urothelial carcinoma is the most common malignant tumor of the lower urinary tract in dogs and is characterized by aggressive behavior, with a high capacity for invasion and metastasis. This study evaluated the expression of EGFR and CD117 proteins in tumors using immunohistochemistry. EGFR and CD117 immunoreactivity was detected in most cases, and both were associated with high-grade tumors. EGFR was independently associated with high histological grade. Furthermore, the simultaneous positivity for EGFR and CD117 was also associated with high histological grade. These results demonstrate frequent co-expression of EGFR and CD117 in canine urothelial carcinoma and their association with aggressive histological features, supporting further investigation of these receptors as potential therapeutic targets.

## 1. Introduction

Canine urothelial carcinoma (UC) is the most common malignant neoplasm of the urinary tract in dogs, accounting for approximately 2% of all spontaneous tumors diagnosed in this species, a proportion similar to that reported in humans. Within the urinary tract, the urinary bladder is the most frequently affected site. Although this neoplasm may also occur in cattle, it is uncommon in other domestic species, such as cats and horses [1,2,3].

It is estimated that approximately 10,000 dogs are affected worldwide each year, with more than 90% of tumors exhibiting highly invasive behavior and metastatic potential. According to the World Health Organization (WHO) staging system, most dogs present with T2 tumors at the time of diagnosis, which are characterized by invasion of the bladder wall, whereas approximately 20% present with T3 disease involving adjacent structures [4,5]. In addition to local invasion, metastatic spread is one of the main factors associated with morbidity and reduced survival. Improvements in therapeutic strategies for controlling the primary tumor have prolonged patient survival; however, this increased longevity has also led to a higher incidence of clinically detectable metastases, maintaining a poor prognosis despite the use of more intensive chemotherapy protocols [6,7,8,9].

Despite significant advances in the clinical and histopathological characterization of this neoplasm, the molecular mechanisms underlying its development and progression remain incompletely understood. The investigation of biomarkers involved in carcinogenesis, particularly those associated with cell signaling pathways, may improve our understanding of tumor biology and support the development of novel diagnostic, prognostic, and therapeutic strategies [10].

Receptor tyrosine kinases comprise a large family of transmembrane proteins consisting of an extracellular ligand-binding domain, a single transmembrane region, and an intracellular domain with catalytic activity [11,12]. Under physiological conditions, ligand binding induces receptor dimerization and autophosphorylation, thereby activating key signaling cascades, including the PI3K/AKT and RAS/MAPK pathways, that regulate cell growth, differentiation, and survival. In many types of cancer, alterations in these mechanisms result in persistent activation of these pathways, promoting uncontrolled proliferation, resistance to apoptosis, and tumor progression. Consequently, RTKs have become major targets for precision oncology, with several tyrosine kinase inhibitors currently incorporated into both human and veterinary cancer therapy [13,14,15,16,17,18].

Among RTKs, the epidermal growth factor receptor (EGFR) is a transmembrane glycoprotein belonging to the ErbB family of RTKs, which includes ErbB-1 (EGFR), ErbB-2 (HER2/neu), ErbB-3 (HER3), and ErbB-4 (HER4) [19]. EGFR plays an important role in cell proliferation, survival, tissue invasion, and angiogenesis, and its activation is associated with the development and progression of various human and canine tumors. In human urothelial carcinomas, high EGFR expression is common and is associated with greater tumor aggressiveness and a poorer prognosis, which has prompted the development/evaluation of EGFR-targeted therapies [20]. In veterinary medicine, although high EGFR expression has been reported in various canine neoplasms, including mammary tumors, gliomas, osteosarcomas, and cutaneous tumors, studies on canine urothelial carcinoma remain relatively scarce [21,22,23]. The available data suggest that this receptor is also expressed in this neoplasm; however, further research is needed to clarify its role in the pathogenesis, progression, and prognosis of the disease, as well as its potential as a biomarker and therapeutic target [24,25].

Another important RTK is KIT (CD117), a transmembrane protein with tyrosine kinase activity, encoded by the KIT proto-oncogene and activated upon binding to stem cell factor (SCF) [26,27]. It is an important member of the type III receptor tyrosine kinase family; other tyrosine kinase receptor molecules include the macrophage colony-stimulating factor receptor CSF1R, Platelet-Derived Growth Factor Receptors Alpha and Beta (PDGFRα/PDGFRβ), and the FLT3 receptor [27]. Activation of this receptor initiates multiple intracellular signaling pathways that regulate cell proliferation, differentiation, migration, survival, and apoptosis [28]. Alterations in KIT signaling have been described in several human and veterinary malignancies and constitute a well-established therapeutic target, particularly in canine mast cell tumors. However, the biological role of KIT in canine urothelial carcinoma remains poorly understood [29]. Although its expression has been demonstrated by immunohistochemistry in a subset of tumors, there is currently no consensus regarding its clinical or prognostic significance, highlighting the need for further investigation [30].

Therefore, the present study aimed to characterize the immunohistochemical expression of EGFR and CD117 in canine urothelial carcinomas and to evaluate their associations with histological grade and other clinicopathological features.

## 2. Materials and Methods

### 2.1. Case Characterization: Clinicopathological Features

This retrospective study included 56 archived samples of bladder tumors from dogs, collected between 2015 and 2025. Samples were retrieved from the archives of the Histology and Anatomical Pathology Laboratory of the University of Trás-os-Montes and Alto Douro (UTAD), Portugal, and the INNO Veterinary Laboratory, Portugal, where they had previously been fixed in 10% neutral buffered formalin and routinely processed for paraffin embedding at the time of the original diagnosis. Clinical information, including age, sex, and breed, was recorded for each animal.

For each case, all available paraffin-embedded blocks and histological sections were retrieved and re-examined. In cases with more than one available tissue block, all blocks were examined on hematoxylin and eosin (H&E)-stained sections to assess overall tumor morphology, and the original histopathological diagnosis was reviewed and confirmed by the study pathologists in accordance with the World Health Organization (WHO) classification criteria [4].

Only cases with a confirmed histopathological diagnosis of canine urothelial carcinoma and sufficient archived material available for immunohistochemical evaluation were included in the study; cases without a confirmed diagnosis or with an insufficient amount of tumor tissue were excluded.

For the histopathological evaluation of canine urothelial carcinomas, two distinct classification systems were applied, as described in Table 1 and Table 2. Histological grade was determined using multiple morphological criteria, including architecture, cellular polarity, nuclear atypia, mitotic activity, and invasion (Table 1). The tumors were graded into low- or high-grade urothelial carcinomas, adapted from [31]. In cases where invasion could not be fully assessed, histological grade was determined primarily based on mitotic activity, nuclear atypia, and cellular/architectural features (loss of cell polarity, absence of the umbrella cell layer, and basal cell layer disorganization), as recommended by Meuten [31].

In addition, tumors were classified according to their growth pattern as papillary or non-papillary, infiltrative or non-infiltrative (Table 2) [4].

### 2.2. Immunohistochemical Analysis of EGFR and CD117

The immunohistochemical expression of EGFR and CD117 was assessed using the NovolinkTM Polymer Detection System (Leica Biosystems^®^, Newcastle, UK). In cases with more than one available tissue block, the most representative block, selected based on the deepest area of invasion and predominant histological features, was used for immunohistochemical evaluation. The histological sections were deparaffinized in xylene, rehydrated in decreasing concentrations of ethanol, and subjected to antigen retrieval in citrate buffer (pH 6.0) for 20 min in a microwave oven. After cooling to room temperature, the slides were washed in phosphate-buffered saline (PBS, pH 7.4). Endogenous peroxidase activity was blocked with Peroxidase Block, followed by blocking of nonspecific binding with Protein Block, both for 5 min. The sections were incubated with the anti-EGFR primary antibody (clone 31G7, mouse monoclonal antibody; Invitrogen, Paisley, Scotland, UK, Ref. 280005, Lot 981392A) and the anti-CD117/c-KIT rabbit polyclonal antibody (Agilent Dako, Glostrup, Denmark; Ref. A4502, Lot 11514957), both at a 1:50 dilution, overnight at 4 °C. After washing in PBS, the slides were incubated with the Post Primary and Novolink Polymer reagents for 30 min each. The reaction was developed with 3,3′-diaminobenzidine (DAB), and the slides were counterstained with Gill’s hematoxylin. Subsequently, they were dehydrated, cleared in xylene, and mounted with Entellan^®^ (Merk KGaA 64271, Darmstadt, Germany)mounting medium. Samples of canine mammary tumors for EGFR and canine mast cell tumors for CD117, with known positivity for these markers, were used as positive controls. Negative controls were obtained by omitting the primary antibody.

### 2.3. Assessment of EGFR and CD117 Immunoexpression

Two independent observers, blinded to clinical and pathological information, evaluated the immunoreactivity of each sample. When their readings diverged, both examiners re-examined the slides together using a double-headed microscope until a consensus score was reached.

For EGFR, only membranous staining was accepted as evidence of immunoexpression. EGFR expression was assigned to one of four categories based on the highest staining intensity observed, an approach adapted from HER-2 interpretation criteria: no staining or staining in fewer than 10% of tumor cells (Score 0, negative); weak/incomplete membranous staining (1+); weak-to-moderate/complete membranous staining (2+); or strong/complete membranous staining (3+). Membranous immunoreactivity in ≥10% of tumor cells (Scores 1+ to 3+) was considered evidence of EGFR expression, whereas EGFR positivity, the threshold used for all subsequent statistical analyses, was defined more strictly as staining of 2+ or 3+ in ≥10% of tumor cells [32,33].

The immunohistochemical expression of CD117 (c-kit) was evaluated semiquantitatively based on the intensity of membrane and/or cytoplasmic staining and the percentage of immunoreactive neoplastic cells, according to criteria adapted from [29,34]. Immunoreactivity was classified into three categories (Score 0, 1+, and 2+). Cases with no staining or weak staining in <10% of neoplastic cells were classified as negative (Score 0). A Score of 1+ was assigned to cases with weak staining in ≥10% of neoplastic cells, or with moderate-to-strong staining in ≥10% to <50% of the neoplastic cells. A Score of 2+ was assigned to cases showing moderate-to-strong staining in ≥50% of the neoplastic cells. For the final analysis of CD117 expression, Scores of 1+ and 2+ (i.e., staining in ≥10% of neoplastic cells) were considered positive, while a Score of 0 was considered negative [29,34].

### 2.4. Statistical Analysis

Statistical analyses were performed using SPSS software (version 31; SPSS Inc., Chicago, IL, USA). Descriptive statistics summarized the clinicopathological characteristics of the study population, with categorical variables presented as absolute numbers and percentages and continuous variables (age) as mean ± SD, median, and range. Interobserver agreement for EGFR and CD117 scoring was quantified using Cohen’s kappa coefficient (κ).

Associations between EGFR expression, CD117 expression, and clinicopathological variables were evaluated using Pearson’s chi-square test or Fisher’s exact test, as appropriate, with exact *p*-values (Fisher-Freeman-Halton) used for tables with small expected cell counts; the Mann–Whitney U test was used for age. Odds ratios (OR) with 95% confidence intervals were calculated for dichotomized marker expression. The relationship between EGFR and CD117 scores was also assessed using Spearman’s correlation coefficient. In light of the dependence between grade and invasion status in the grading system used, the univariate association between EGFR/CD117 expression and infiltrative growth pattern was not performed.

Multivariate logistic regression, adjusting for age, sex, and breed, was used to identify independent predictors of histological grade. Histological grade (dependent variable) was coded as high-grade (1) versus low-grade (0, reference); sex was coded as male (1) versus female (0, reference); breed was coded as purebred (1) versus mixed breed (0, reference); and age was entered as a continuous variable (per year). Cases with missing data on age or breed were excluded from the multivariable model. Because several covariate combinations resulted in quasi-complete separation, multivariable models were estimated using Firth’s bias-reduced (penalized) logistic regression. Due to collinearity, as concurrent positivity is mathematically derived from both markers, a separate multivariable model, adjusting for the same covariates, was constructed to evaluate concurrent EGFR/CD117 positivity as a combined predictor of histological grade. All statistical tests were two-sided, and *p*-values < 0.05 were considered statistically significant.

## 3. Results

### 3.1. Clinicopathological Findings

A total of 56 dogs, representing 13 breeds, were included in the study. Mixed-breed dogs predominated, accounting for 37 cases (66.1%). The Labrador Retriever was the second most common breed with 4 dogs (7.1%), followed by the Poodle with 3 dogs (5.4%) and the Miniature Pinscher with 2 dogs (3.6%). The remaining breeds, specifically the Chihuahua, Chow Chow, Estrela Mountain Dog, German Shepherd, Jack Russell Terrier, Pointer, Samoyed, Schnauzer, and Siberian Husky, were each represented by a single dog (1.8%). Breed information was unavailable for one dog (1.8%).

Regarding sex distribution, the cohort included 29 males (51.8%) and 27 females (48.2%), demonstrating a slight male predominance. Age data were available for 52 animals. Age ranged from 4 to 19 years, with a mean of 10.9 ± 3.22 years, a median of 11 years, and a 95% confidence interval (CI) for the mean spanning 10.03 to 11.78 years.

Among the 56 urothelial carcinomas evaluated, high-grade tumors were the most prevalent, representing 41 cases (73.2%), whereas 15 tumors (26.8%) were classified as low-grade (Table 3). Regarding histological growth patterns, papillary carcinomas were slightly more frequent than non-papillary carcinomas, accounting for 33 (58.9%) and 23 (41.1%) cases, respectively. With respect to invasion status, infiltrative tumors predominated, representing 39 cases (69.6%), whereas 15 tumors (26.8%) were classified as non-infiltrative (Table 3). In two cases, invasion could not be fully assessed.

### 3.2. Immunohistochemical Expression

#### 3.2.1. EGFR

In histologically normal urothelium adjacent to the tumor, EGFR expression was predominantly restricted to the basal cell layer and showed weak cytoplasmic immunoreactivity. In canine urothelial carcinomas, EGFR immunostaining was observed in neoplastic cells, with membrane, cytoplasmic, or combined staining patterns and varying intensities ranging from absent to strong. Only membrane staining was considered in determining EGFR positivity, as described in Section 2.

Interobserver agreement for EGFR scoring was κ = 0.914 (91.5% raw agreement), involving adjacent intensity categories (e.g., 1+/2+ or 2+/3+); these cases were resolved by consensus review using a double-headed microscope.

The staining pattern was distributed as follows (Figure 1 and Figure 2): 5 cases (8.9%) were classified as score 0, showing no membranous staining or membranous staining in <10% of neoplastic cells; 11 cases (19.6%) showed weak and/or incomplete membranous staining (score 1+); 21 cases (37.5%) showed weak-to-moderate, complete membranous staining (score 2+); and 19 cases (33.9%) showed strong, complete membranous staining (score 3+). According to the predefined positivity criterion (membranous staining scored as 2+ or 3+ in ≥10% of neoplastic cells), 40 of 56 tumors (71.4%) were classified as EGFR-positive, whereas 16 of 56 (28.6%) were classified as EGFR-negative (Figure 1).

#### 3.2.2. CD117

Beyond tumor cells, CD117 staining was consistently observed in mast cells and macrophages, which served as internal positive controls.

Interobserver agreement for CD117 scoring, assessed using Cohen’s kappa coefficient, was κ = 1.0 (100% raw agreement), with no discordant cases.

Immunohistochemical analysis of CD117 was evaluated across 56 tumor cases based on staining intensity and cellular extent. A complete absence of immunoreactivity was observed in 10 cases (17.9%). Weak, moderate, and strong staining intensities were detected in 18 (32.1%), 18 (32.1%), and 10 (17.9%) cases, respectively. In terms of cellular extent, 28 cases (50.0%) showed CD117 expression in 50% or more of tumor cells, whereas 18 cases (32.1%) showed expression in less than 50% of tumor cells (Figure 3 and Figure 4). After applying the combined classification system, 20 cases (35.7%) were classified as Score 2+, and 36 cases (64.3%) as Score 1+ or Score 0.

Considering the previously established positivity threshold (≥10% of immunostained neoplastic cells), 46 of the 56 cases (82.1%) were classified as positive for CD117 expression (Scores 1+ and 2+), while 10 cases (17.9%) were classified as negative (Score 0), (Figure 3).

#### 3.2.3. Association of EGFR, CD117 and Concurrent Positivity with Clinicopathological Characteristics

EGFR positivity was significantly associated with a high histological grade (*p* < 0.0001). No significant association was found with papillary architecture (*p* = 0.549) or other clinical variables.

CD117 positivity was also significantly associated with histological grade (*p* = 0.016), with CD117-positive tumors being more frequently high-grade (37/46, 80.4%) than CD117-negative tumors (4/10, 40.0%). No statistically significant association was noted with papillary architecture (*p* = 0.172) or other variables (Table 4).

Regarding the relationship between EGFR and CD117 co-expression and histopathological variables, the three co-expression categories (both markers positive, only one marker positive, and both markers negative) were significantly associated with histological grade (*p* < 0.0001). No significant associations were found with papillary architecture, sex, breed, or age (*p* > 0.15). Tumors with concurrent EGFR and CD117 positivity were markedly more likely to be high-grade (35/38, 92.1%) than tumors with only one marker positive (3/10, 30.0%) or those with both markers negative (3/8, 37.5%), (Figure 5).

**Figure 5:**
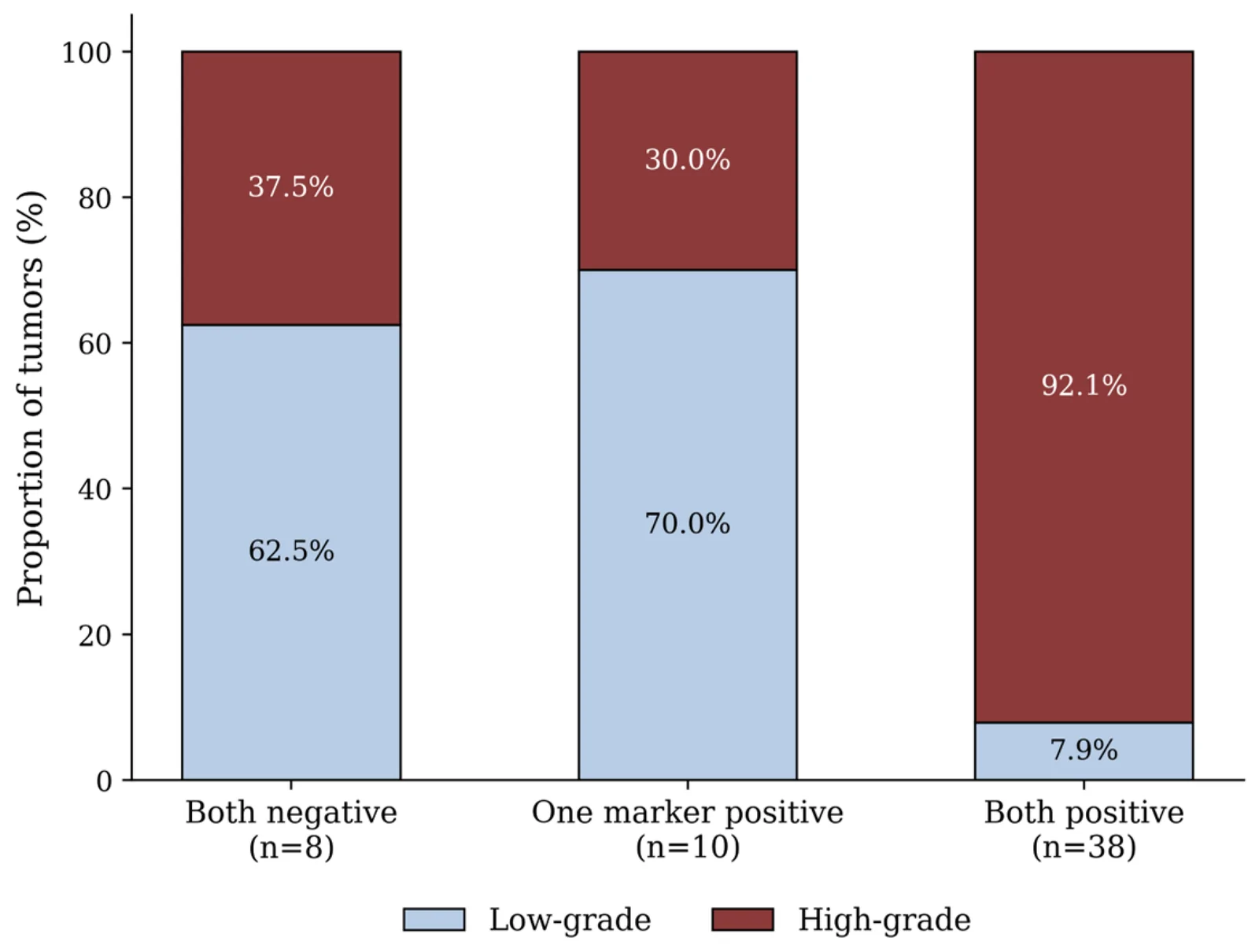
Histological grade distribution according to EGFR/CD117 co-expression in canine urothelial carcinomas.

#### 3.2.4. Multivariate Predictors of Histological Grade

To determine whether EGFR and CD117 positivity were independently associated with histological grade, a multivariate logistic regression model was performed, adjusting for age, sex, and breed (purebred vs. mixed-breed). EGFR positivity remained independently associated with a high histological grade (*p* = 0.0024), consistent with the univariate analysis. In contrast, CD117 positivity was no longer significantly associated with histological grade in the multivariate model (*p* = 0.509) (Table 5).

In a separate multivariate model evaluating concurrent EGFR/CD117 positivity as a combined predictor, adjusting for age, sex, and breed, only concurrent positivity remained independently associated with high histological grade (OR = 14.27, 95% CI: 3.05–66.78, *p* = 0.0007; *n* = 51 dogs with complete covariate data).

#### 3.2.5. Relationship Between EGFR and CD117 Expression

A significant positive correlation was observed between EGFR and CD117 staining scores (ρ = 0.30, *p* = 0.025), indicating that higher EGFR immunoexpression tends to co-occur with higher CD117 labeling, (Figure 6).

**Figure 6:**
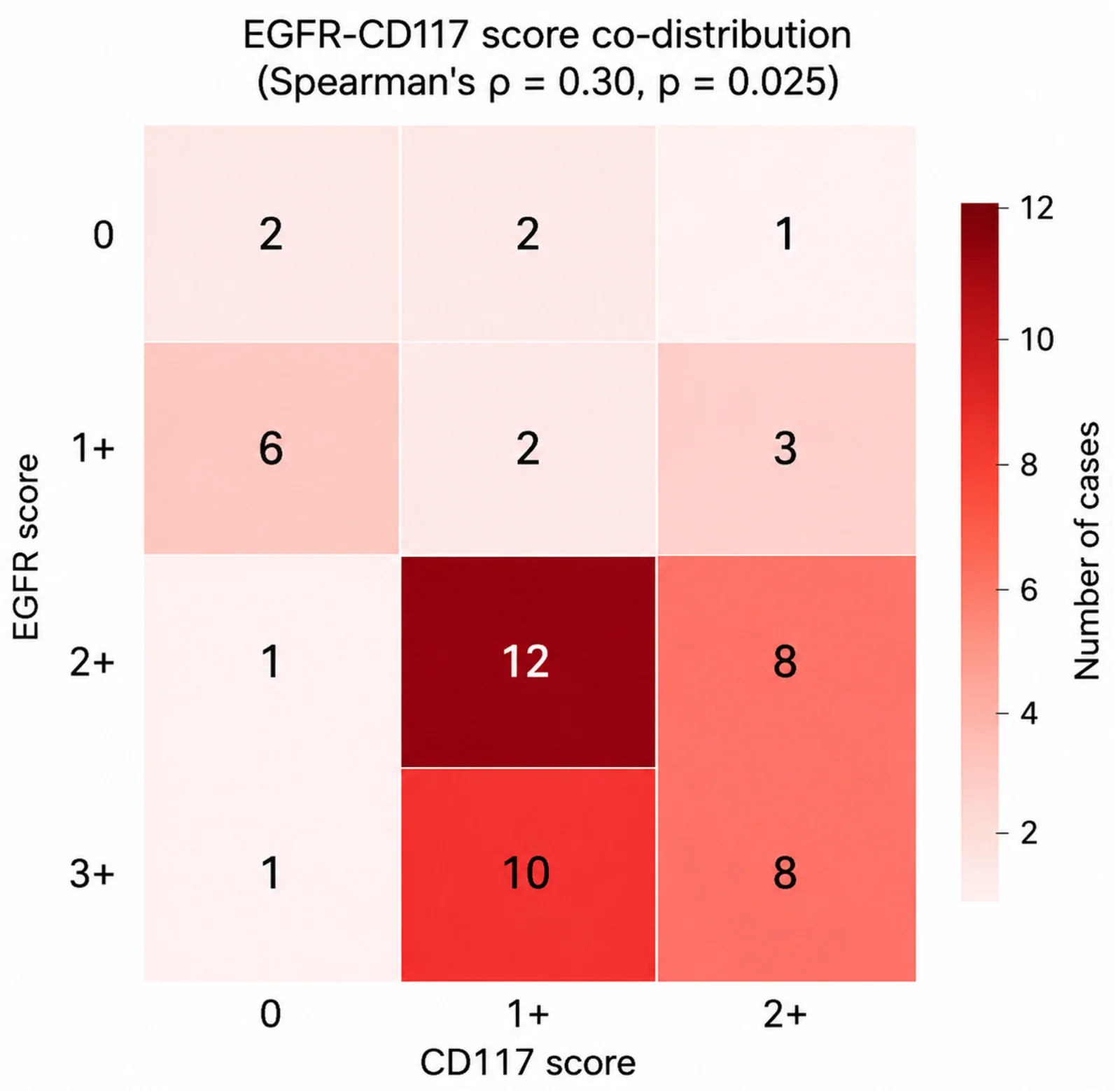
Heatmap of the co-distribution of EGFR and CD117 immunostaining scores among 56 canine urothelial carcinomas.

When immunoexpression was dichotomized as negative or positive, overall, 38 of 56 tumors (67.9%) showed co-expression of EGFR and CD117. Among EGFR-positive tumors, 38 of 40 (95.0%) were concurrently CD117-positive, whereas among EGFR-negative tumors, only 8 of 16 (50.0%) were CD117-positive. A significant positive association was observed between EGFR positivity and CD117 positivity (*p* < 0.001, OR = 19.00, 95% CI: 3.38–106.84), (Figure 7).

## 4. Discussion

Canine urothelial carcinoma is the most common malignant neoplasm of the lower urinary tract in dogs and is characterized by highly aggressive biological behavior, marked by early invasion of the bladder wall and a high metastatic potential [1,35,36]. Despite advances in the histopathological characterization of this neoplasm, there are still few molecular biomarkers with validated diagnostic, prognostic, and therapeutic utility in veterinary medicine. In this context, the present study evaluated the immunohistochemical expression of two tyrosine kinase receptors, EGFR and CD117, seeking to clarify their association with histopathological characteristics of tumor aggressiveness.

Regarding clinical characteristics, the mean age of the dogs was 11 years, a finding consistent with the literature, which describes urothelial carcinoma as a neoplasm typically seen in geriatric animals [37]. A slight predominance of males was also observed, in contrast to most published studies, which report that females are more frequently affected [38]. This difference may reflect the small sample size or characteristics specific to the studied population. However, since sex was not significantly associated with either EGFR (*p* = 0.240) or CD117 expression (*p* = 0.171), this slight sex imbalance observed in our cohort did not confound the immunohistochemical findings. Similarly, the high proportion of mixed-breed dogs should be interpreted with caution, as it likely reflects the distribution of the Portuguese canine population rather than a specific breed predisposition [38].

Histologically, there was a predominance of high-grade carcinomas (73.2%). Regarding invasion status, infiltrative growth was identified in 39 of 54 evaluable cases (72.2%), a lower proportion than the >90% invasion rates reported in some previous studies of canine urothelial carcinoma [37,39]. This difference may reflect variation in case selection, diagnostic timing, or grading/classification criteria across studies, and should therefore be interpreted with caution rather than as directly concordant with these earlier reports.

EGFR is a transmembrane receptor of the ErbB family that activates signaling pathways involved in cell proliferation, differentiation, migration, and apoptosis [19,40,41].

In the present study, EGFR immunoreactivity was observed in 91.1% of the canine urothelial carcinomas evaluated, while 71.4% met the defined criteria for EGFR positivity. This finding is consistent with previous evidence of EGFR expression in canine urothelial carcinoma. Hanazono et al. reported high EGFR expression in 72% (18/25) of canine transitional cell carcinomas, supporting the frequent expression of this receptor in canine bladder tumors [20]. Similar findings have also been reported in human urothelial carcinoma, where EGFR expression is observed in 46% to 74% of cases, including approximately 70% of upper urinary tract urothelial carcinomas [42,43]. Moreover, canine bladder tumors exhibit significantly higher EGFR expression than normal urothelium and polypoid cystitis, supporting its potential value as a diagnostic biomarker for urothelial neoplasia [20].

Regarding staining intensity, most positive tumors in the present study showed moderate to strong immunoreactivity consistent with previous canine studies [20,44]. In contrast, studies in human urothelial carcinoma have reported a lower proportion of tumors with high EGFR expression, for example, in 33 of 126 cases [20] and 20 of 56 analyzed tumors [45].

Beyond its diagnostic value, EGFR may also represent a promising biomarker of tumor aggressiveness and prognosis. In the present study, high EGFR expression was significantly associated with histological grade; however, this association was not observed in a study evaluating 25 canine UCs [20]. In human UC, high expression of this receptor has been consistently associated with high histologic grade, advanced tumor stage, muscle invasion, tumor necrosis, lymphovascular invasion, disease progression, and shorter survival [46,47].

In addition to urothelial carcinoma, high EGFR expression has also been associated with greater aggressiveness in other canine neoplasms, such as cutaneous squamous cell carcinoma [23], canine mammary carcinoma [21], and canine nasal carcinomas [48], suggesting the prognostic value of this biomarker. Comparable associations have been described in human oncology, namely breast carcinomas and squamous cell carcinomas of the oral cavity [49].

Given the high frequency of EGFR expression observed in our cohort and its strong association with aggressive tumor features, this receptor represents a promising candidate for targeted therapy in canine oncology [50]. Blocking the ErbB/MAPK cascade has already been proposed for canine bladder cancer [24], and near-infrared photoimmunotherapy using a canine-specific anti-EGFR antibody has shown selective antitumor efficacy in preclinical models of canine urothelial carcinoma [25]. Small-molecule tyrosine kinase inhibitors and anti-EGFR monoclonal antibodies, already widely used in human precision oncology, also warrant prospective evaluation in dogs [51,52,53].

CD117 (c-KIT) is a transmembrane tyrosine kinase receptor encoded by the *KIT* proto-oncogene, playing a key role in regulating cell proliferation, differentiation, survival, migration, and apoptosis. Under physiological conditions, c-KIT is expressed on the plasma membrane as a monomer and is activated by binding its ligand, Stem Cell Factor (SCF), which induces receptor dimerization and autophosphorylation of tyrosine residues. This process activates various intracellular signaling pathways, including PI3K/AKT, RAS/MAPK, and JAK/STAT, which regulate cell growth and survival. Abnormal activation of c-KIT can occur through activating mutations, gene amplification, high expression, or SCF-dependent autocrine/paracrine activation mechanisms involved in the oncogenesis of various neoplasms [54,55,56].

In the present study, CD117 expression was observed in 82% of canine urothelial carcinomas, a value higher than Setyo et al., who reported positivity in 15% (3/20) [57], but similar to studies in human UC [29]. These differences may be attributed to methodological factors, such as sample size, antibody clone, immunohistochemical protocols, criteria for interpreting immunostaining, and biological heterogeneity of the populations studied.

According to our results, CD117 positivity was more frequent in high-grade tumors, as the analysis used binary positivity. This finding suggests that CD117 expression is associated with a more aggressive biological phenotype. Although few studies have evaluated this association in canine urothelial carcinoma, studies in other canine neoplasms demonstrate that the biological significance of CD117 varies by tumor type. In soft-tissue sarcomas, for example, Dell’Aere et al. observed no consistent association between CD117 expression and histological grade, suggesting that this receptor is not a universal marker of aggressiveness [58]. In contrast, in canine mammary tumors, CD117 expression was observed predominantly in malignant neoplasms, whereas benign tumors showed reduced or absent expression, suggesting that activation of this pathway may be involved in malignant transformation [59].

In human medicine, the association between CD117 expression and high histological grade in urothelial carcinoma remains controversial. Some studies have demonstrated an association between c-KIT expression and clinically and pathologically aggressive features, including high-grade tumors and more advanced disease. In contrast, other authors have not observed a significant association between CD117 expression and tumor progression, suggesting that markers such as HER2 may have greater prognostic value in high-grade urothelial carcinomas [29,30]. These discrepancies likely reflect methodological differences among the studies and the biological heterogeneity of the disease, underscoring the need for further research to clarify the role of c-KIT in human and canine urothelial carcinoma.

From a biological perspective, c-KIT has also been implicated in the epithelial–mesenchymal transition (EMT), a fundamental mechanism for invasion and metastatic spread. Activation of this pathway increases expression of mesenchymal markers, such as N-cadherin, vimentin, Snail, and Twist, while simultaneously reducing E-cadherin expression, thereby promoting loss of cell adhesion, increased motility, and tumor invasion [54,60,61].

In addition to its potential prognostic value, c-KIT represents an important therapeutic target. Tyrosine kinase inhibitors, such as imatinib and masitinib, have demonstrated clinical efficacy in various neoplasms dependent on KIT activation. In veterinary medicine, masitinib is approved for the treatment of canine mast cell tumors harboring activating mutations in KIT, highlighting the clinical relevance of this receptor as a therapeutic target [62]. Although the therapeutic significance of CD117 in canine urothelial carcinoma has not yet been established, the high frequency of expression observed in the present study, combined with its association with high-grade tumors, suggests that c-KIT may represent a marker associated with high histological grade in univariable analysis and a promising target for precision therapies, warranting further studies to validate its clinical applicability [63].

To the best of our knowledge, this is the first study to simultaneously evaluate the immunohistochemical expression of EGFR and CD117 in canine urothelial carcinoma. Although direct comparison with the veterinary literature is not possible, previous studies in human urothelial carcinoma have shown that both receptors are individually associated with high histologic grade and advanced tumor stage [64]. In the present study, the significant association between EGFR and CD117 expression, together with the predominance of high-grade tumors among cases positivity for both markers, may reflect co-expression of these receptor tyrosine kinases during canine urothelial carcinogenesis. Although a direct interaction between EGFR and c-KIT has not been demonstrated, both receptors activate common downstream pathways, including PI3K/AKT and RAS/MAPK, supporting the hypothesis that co-expression may be relevant within interconnected RTK signaling networks. This interpretation is supported by the concept of the RTK interactome, which proposes that receptor tyrosine kinases function as an extensive, interconnected signaling network in which interactions between different RTKs can amplify, modify, or compensate for downstream signaling. Such receptor crosstalk has been implicated in tumor progression and therapeutic response in several human malignancies, even in the absence of direct evidence for specific receptor pairs, such as EGFR and c-KIT [16,65]. As the present study is based solely on immunohistochemical expression, without functional assays of receptor phosphorylation or downstream pathway activation, these interpretations remain hypothesis-generating and warrant confirmation in future mechanistic studies.

Collectively, these findings reinforce the biological relevance of EGFR and CD117 in canine urothelial carcinoma and support further studies to determine their prognostic value and potential as therapeutic targets. Moreover, because both receptors are established therapeutic targets in other malignancies, their co-expression warrants further investigation of combined RTK-targeted therapeutic strategies in canine urothelial carcinoma. In human oncology, the efficacy of single-agent tyrosine kinase inhibitors is often limited by the emergence of acquired resistance, which can arise from compensatory activation of alternative receptor tyrosine kinase (RTK) pathways and the maintenance of downstream survival signaling. Such pathway redundancy provides a strong rationale for multi-targeted therapeutic strategies that simultaneously suppress complementary oncogenic signaling networks. Accordingly, combining agents that target different RTKs or using multi-kinase inhibitors may help limit adaptive escape mechanisms and enhance antitumor activity. This concept may be particularly relevant to canine urothelial carcinoma, in which multiple RTKs, including EGFR, KIT, PDGFRs and VEGFRs, may be co-expressed or activated [57,63]. KIT phosphorylation has been demonstrated in a subset of primary canine urothelial carcinomas, although the multi-targeted tyrosine kinase inhibitor toceranib did not show a significant direct antiproliferative effect in the canine UC cell lines tested [63]. These findings provide a rationale for investigating whether simultaneous targeting of EGFR and complementary RTK pathways, including KIT-associated signaling, could improve therapeutic efficacy in dogs with high-grade infiltrative urothelial carcinoma [24]. However, such combination strategies remain investigational and require prospective preclinical and clinical validation. Beyond their potential veterinary benefit, these studies could further strengthen the use of naturally occurring canine urothelial carcinoma as a comparative model for evaluating resistance mechanisms and precision-targeted therapeutic strategies relevant to human cancer [63].

Several limitations should be acknowledged. The retrospective nature of the study, the relatively limited sample size, the absence of follow-up and survival data, and the lack of molecular assessment of EGFR and KIT alterations preclude definitive conclusions regarding prognostic significance or therapeutic response. Furthermore, most samples were small biopsy specimens, often lacking complete representation of the bladder wall layers, limiting assessment of muscularis propria and adventitial invasion and evaluation of tumor emboli. Additionally, immunohistochemical expression of EGFR and KIT does not establish receptor activation, mutation status, or functional dependence, thereby limiting the therapeutic interpretation of these findings.

Prospective studies with larger sample sizes, including survival analyses and molecular characterization, are needed to clarify their prognostic and predictive value and to determine their utility in selecting targeted therapies.

Overall, these findings support the hypothesis that frequent co-expression of EGFR and CD117 is associated with higher histological grade in canine urothelial carcinoma, highlighting these receptors as candidates for further prognostic and therapeutic investigation.

## 5. Conclusions

EGFR and CD117 are frequently expressed in canine urothelial carcinoma and are associated with histopathological features of tumor aggressiveness. EGFR remained independently associated with high histological grade in multivariate analysis, whereas CD117 was associated with high histological grade in univariate analysis but not in multivariate analysis. The striking 67.9% (38 of 56) co-expression rate suggests that EGFR and CD117 are frequently co-expressed during urothelial carcinogenesis. These results provide new insights into the biology of canine urothelial carcinoma and support the conduct of further prospective studies that integrate molecular and functional analyses to clarify the prognostic and biological significance of EGFR and CD117 expression. Although their co-expression identifies a potentially relevant biological phenotype, the present results alone do not demonstrate receptor activation or functional dependence and therefore do not provide sufficient evidence to support dual-target therapy. Instead, they provide a basis for future studies to determine whether simultaneous targeting of EGFR- and KIT-related pathways is therapeutically relevant. In this context, invasive canine bladder cancer may also represent a valuable comparative model for investigating multi-kinase-targeted approaches relevant to precision oncology.

## Figures and Tables

**Figure 1:**
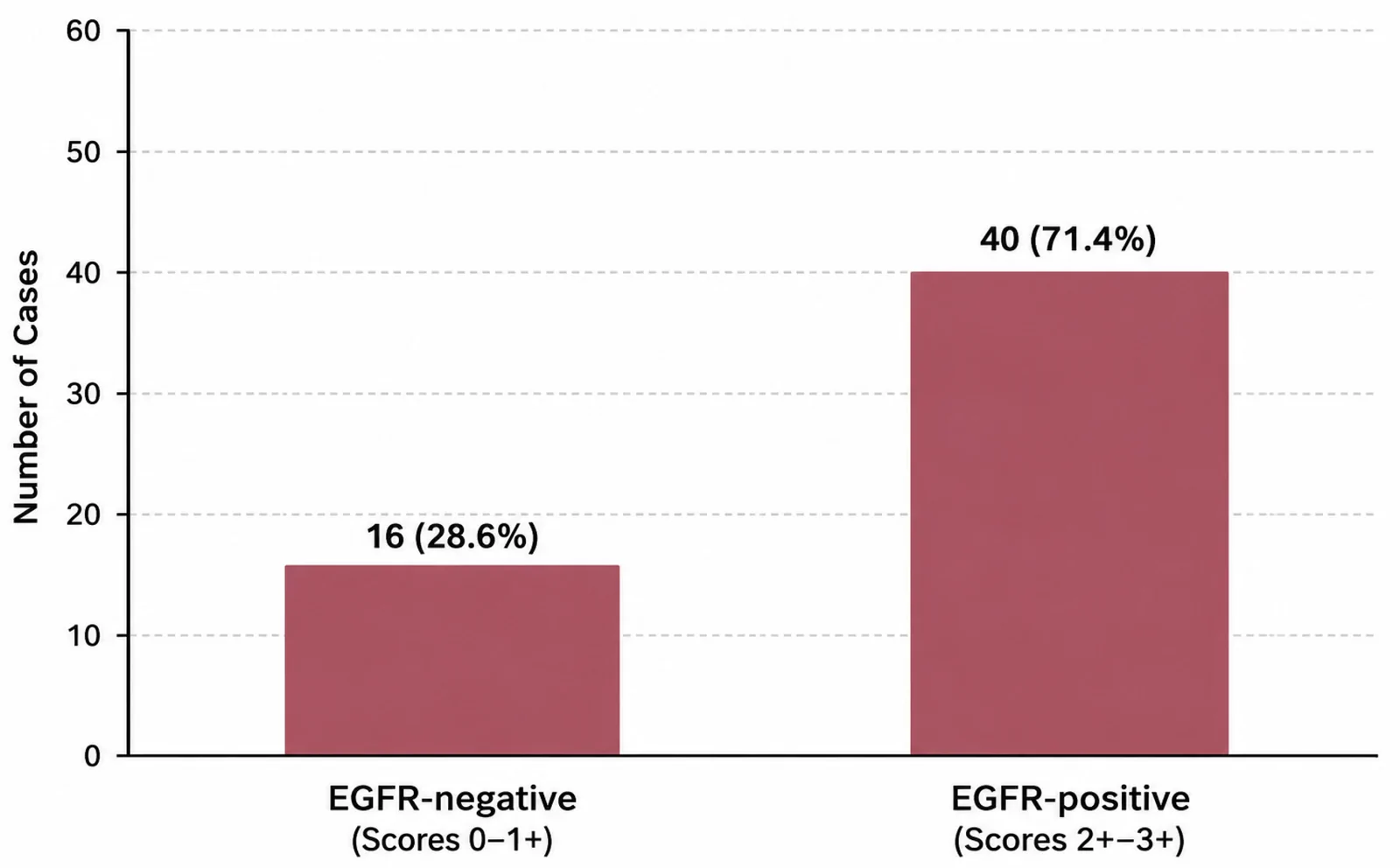
Distribution of EGFR immunohistochemical positivity (negative vs. positive) in canine urothelial carcinoma.

**Figure 2:**
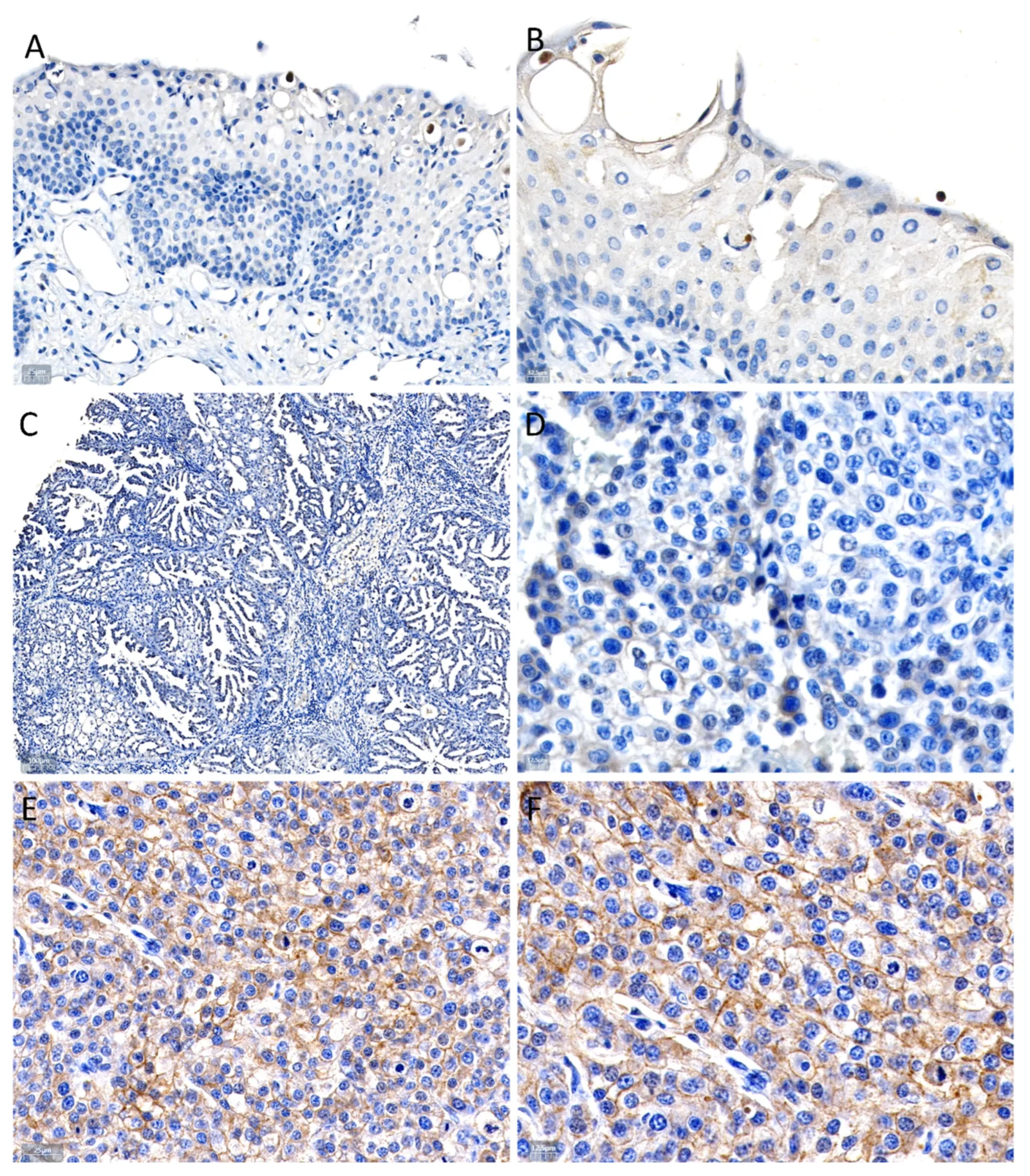
Immunohistochemical expression of EGFR in canine urothelial carcinoma. (**A**) Low-grade urothelial carcinoma showing negative to weak EGFR immunostaining. Scale bar: 25 μm. (**B**) Higher magnification of (**A**). Scale bar: 12.5 μm. (**C**) High-grade papillary urothelial carcinoma with EGFR score 1+, classified as negative according to the study-defined cut-off. Scale bar: 100 μm. (**D**) Urothelial carcinoma showing predominantly weak-to-moderate, complete membrane EGFR immunostaining (score 2+). Scale bar: 12.5 μm. (**E**) High-grade urothelial carcinoma showing strong and complete membrane EGFR immunostaining (score 3+). Scale bar: 25 μm. (**F**) Higher magnification of (**E**). Scale bar: 12.5 μm.

**Figure 3:**
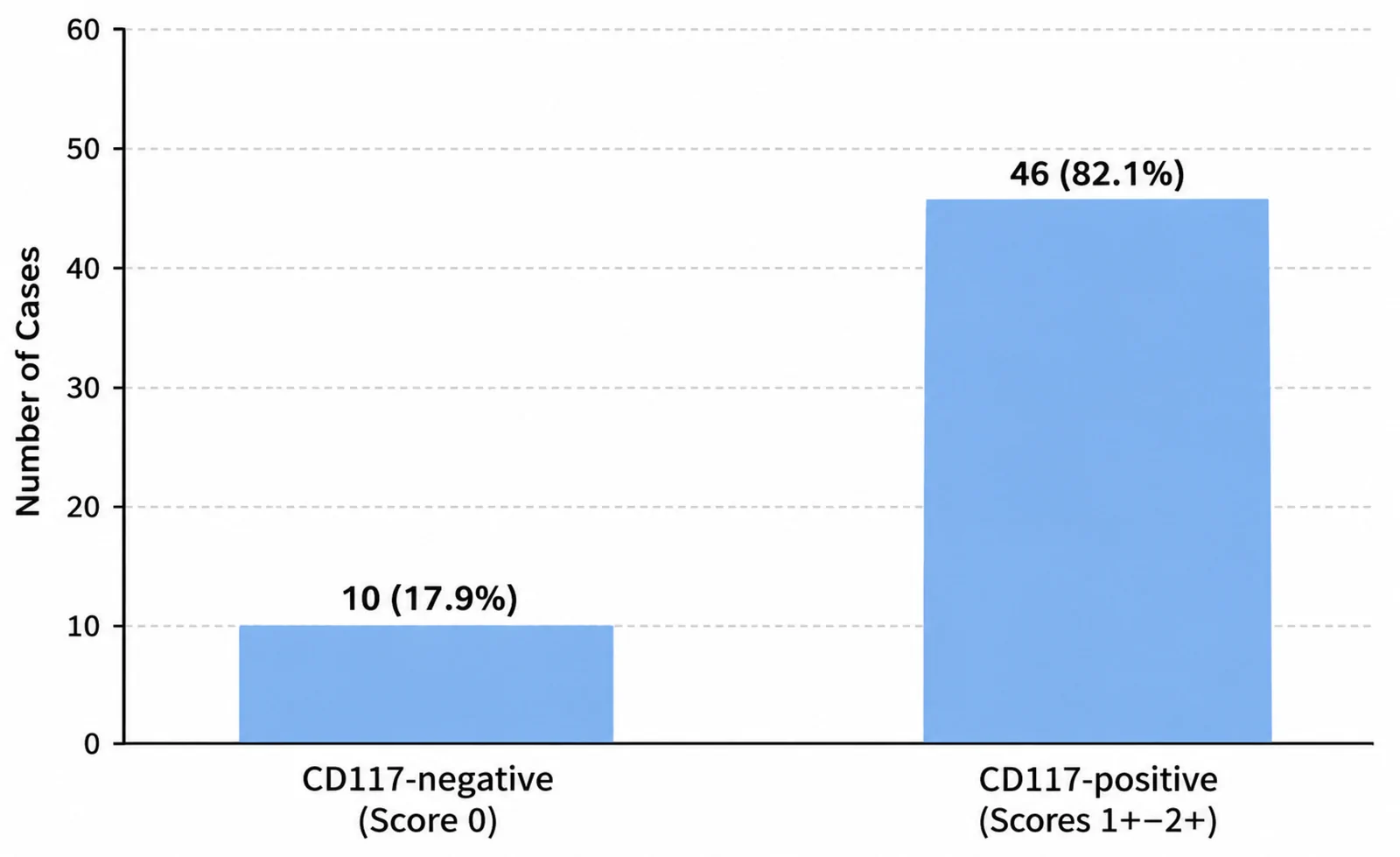
Distribution of CD117 immunohistochemical positivity (negative vs. positive) in canine urothelial carcinoma.

**Figure 4:**
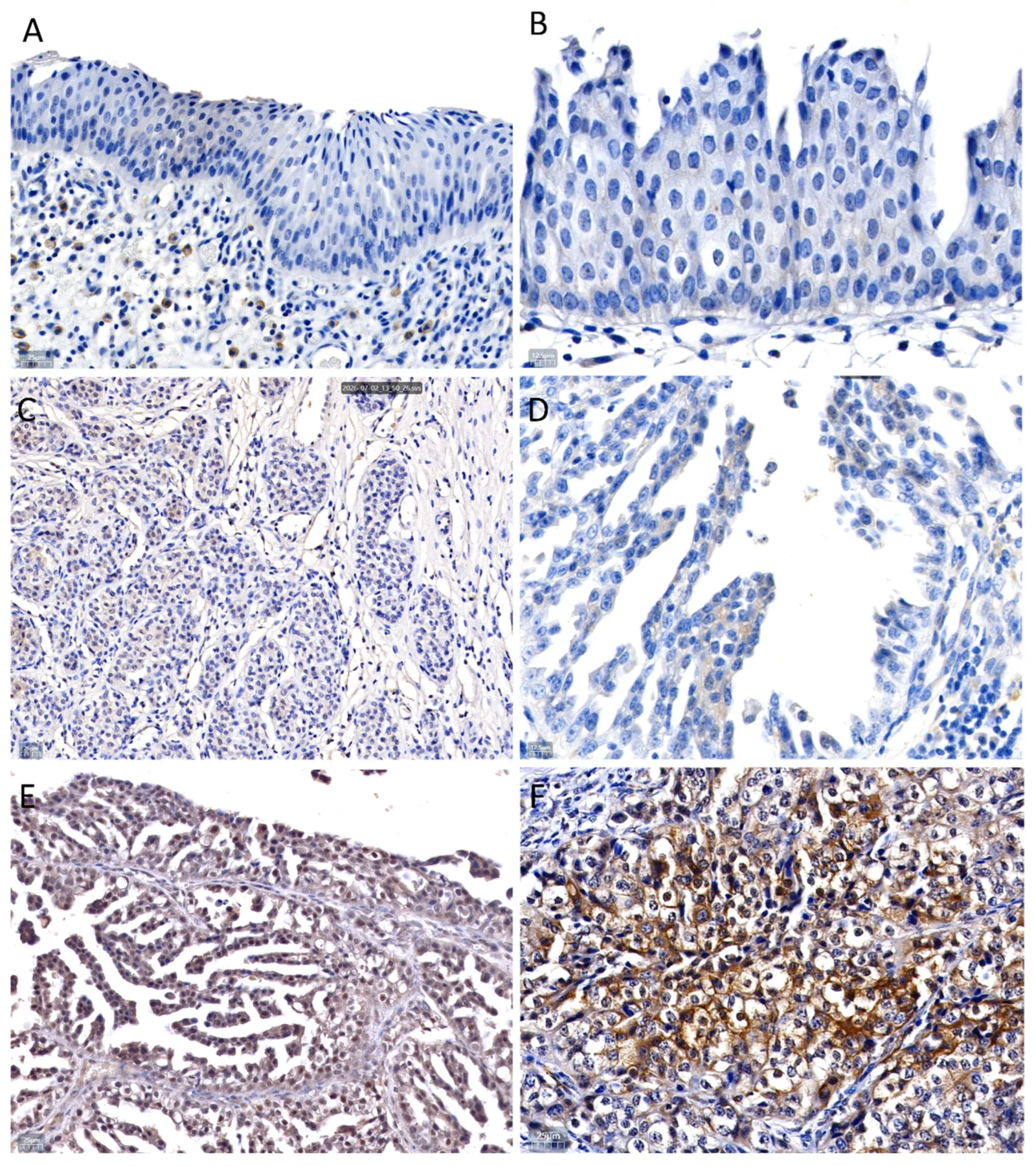
Immunohistochemical expression of CD117 (c-KIT) in canine urothelial carcinoma. (**A**) Low-grade urothelial carcinoma showing negative CD117 immunostaining (score 0); note positive immunostaining in mast cells within the lamina propria, serving as an internal positive control. Scale bar: 25 μm. (**B**) Low-grade urothelial carcinoma showing negative CD117 immunostaining (score 0). Scale bar: 12.5 μm. (**C**) High-grade, non-papillary, invasive urothelial carcinoma showing weak CD117 immunostaining in ≥10% of neoplastic cells (score 1+). Scale bar: 25 μm. (**D**) Urothelial carcinoma showing moderate CD117 immunostaining in ≥50% of neoplastic cells (score 2+). Scale bar: 12.5 μm. (**E**) High-grade urothelial carcinoma showing moderate-to-strong CD117 immunostaining in ≥50% of neoplastic cells (score 2+). Scale bar: 25 μm. (**F**) High-grade urothelial carcinoma showing strong CD117 immunostaining in ≥50% of neoplastic cells (score 2+). Scale bar: 25 μm.

**Figure 7:**
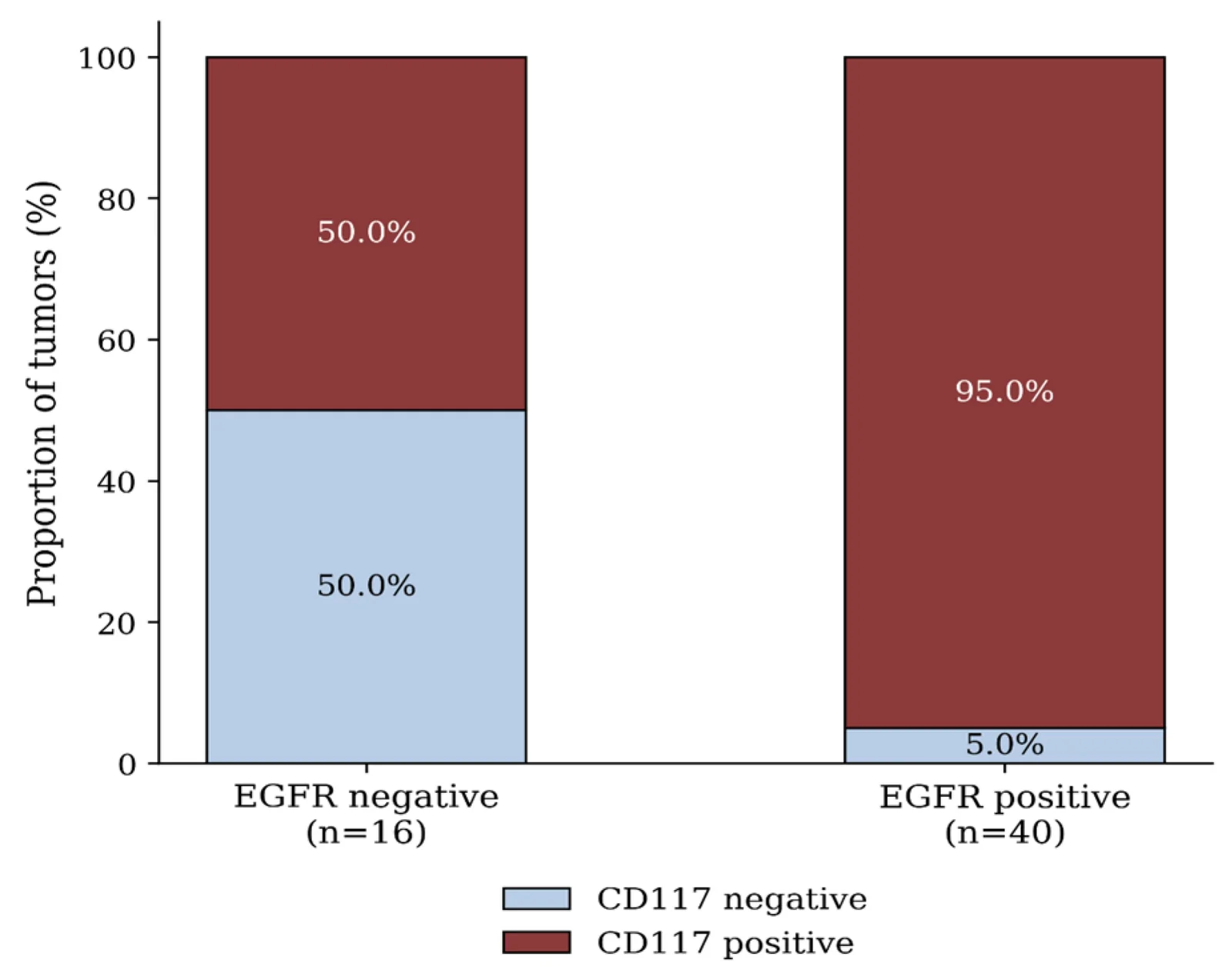
Association between EGFR and CD117 immunohistochemical positivity in canine urothelial carcinoma.

**Table 1:** Histological grading criteria used for the evaluation of canine urothelial carcinoma, adapted from [31].

Histological Grade	Histopathological Characteristics
Low grade	Organized urothelial proliferation, mild to moderate cytological atypia, preserved cellular polarity, rare mitotic figures, and absence of invasion.
High grade	Disorganized urothelial architecture, loss of cellular polarity, marked nuclear atypia, numerous mitotic figures, prominent nucleoli, and evidence of stromal and/or muscular invasion.

**Table 2:** Histopathological classification of urothelial carcinoma according to papillary architecture and invasion status [4].

Histological Classification	Papillary	Non-Papillary
Non- infiltrative	Exophytic papillary growth supported by fibrovascular cores lined by neoplastic urothelial cells. Tumor cells remain confined to the urothelium, with no invasion of the lamina propria or muscular layer.	Flat lesion composed of cytologically malignant urothelial cells confined to the urothelium, without stromal invasion; corresponds to carcinoma in situ.
Infiltrative	Papillary architecture associated with invasion through the fibrovascular core into the lamina propria and, frequently, the muscularis propria. In advanced cases, extension through the adventitia and metastatic spread may occur.	Flat, plaque-like, nodular, or raised lesions lacking papillary architecture. These lesions are frequently ulcerated and characterized by invasion of the bladder wall, marked cytological atypia, and high metastatic potential.

**Table 3:** Clinicopathological characteristics of canine urothelial carcinomas.

Variable	Category	*n* (%)
Sex	Male	29 (51.8%)
	Female	27 (48.2%)
Age (years) ^1^	Mean ± SD (median)	10.9 ± 3.22 (11)
Breed	Mixed breed	37 (66.1%)
	Labrador Retriever	4 (7.1%)
	Poodle	3 (5.4%)
	Miniature Pinscher	2 (3.6%)
	Other breeds ^2^	9 (16.1%)
	Not recorded	1 (1.8%)
Histological grade	High-grade	41 (73.2%)
	Low-grade	15 (26.8%)
Growth pattern	Papillary	33 (58.9%)
	Non-papillary	23 (41.1%)
Invasion status ^3^	Infiltrative	39 (69.6%)
	Non-infiltrative	15 (26.8%)

^1^ Age data were available for 52/56 dogs (95% CI for the mean: 10.03–11.78 years). ^2^ Chihuahua, Chow Chow, Estrela Mountain Dog, German Shepherd, Jack Russell Terrier, Pointer, Samoyed, Schnauzer, and Siberian Husky (1 dog each). ^3^. In 2 cases, assessment of invasion was limited by incomplete representation of the bladder wall in the biopsy specimen.

**Table 4:** Association of EGFR and CD117 positivity with clinicopathological characteristics.

Clinicopathological Variable	EGFR		CD117	
	OR (95% CI)	*p*-Value	OR (95% CI)	*p*-Value
High histological grade	19.80 (4.52–86.81)	<0.0001	6.17 (1.43–26.55)	0.016
Papillary architecture	1.67 (0.52–5.37)	0.549	0.30 (0.06–1.56)	0.172
Male sex	2.25 (0.69–7.42)	0.240	3.03 (0.70–13.23)	0.171
Purebred (vs. mixed breed)	0.80 (0.24–2.69)	0.761	2.34 (0.45–12.35)	0.467
Age (per year)	Not applicable *	0.843	Not applicable *	0.730

* Odds ratios (ORs) and 95% confidence intervals (CIs) are not applicable because age was analyzed as a continuous variable using the Mann–Whitney U test.

**Table 5:** Multivariate logistic regression analysis of histological grade and clinicopathological risk factors *(n* = 51 dogs with complete covariate data).

High-Grade Tumor Variable	OR (95% CI)	*p*-Value
Age (per year)	1.08 (0.84–1.39)	0.541
Male sex	0.88 (0.16–4.98)	0.888
Purebred (vs. mixed breed)	0.98 (0.16–5.82)	0.982
EGFR positivity	27.18 (3.22–229.41)	0.0024
CD117 positivity	0.43 (0.03–5.36)	0.509

## Data Availability

The original contributions presented in this study are included in the article. Further inquiries can be directed to the corresponding authors.
